# Allotransplantation Is Associated With Exacerbation of CD8 T-Cell Senescence: The Particular Place of the Innate CD8 T-Cell Component

**DOI:** 10.3389/fimmu.2021.674016

**Published:** 2021-07-21

**Authors:** Lauren Daniel, Marion Tassery, Clara Lateur, Antoine Thierry, André Herbelin, Jean-Marc Gombert, Alice Barbarin

**Affiliations:** ^1^ Inserm U1082, Poitiers, France; ^2^ Université de Poitiers, Poitiers, France; ^3^ Service de Néphrologie, Hémodialyse et Transplantation, CHU de Poitiers, Poitiers, France; ^4^ Service d’Immunologie et Inflammation, CHU de Poitiers, Poitiers, France; ^5^ CHU de Poitiers, Poitiers, France

**Keywords:** innate memory CD8(+) T-cells, NK-like T-cells, allotransplantation, senescence, immune memory, innateness gradient

## Abstract

Immunosenescence is a physiological process that is associated with changes in the immune system, particularly among CD8 T-cells. Recent studies have hypothesized that senescent CD8 T-cells are produced with chronologic age by chronic stimulation, leading to the acquisition of hallmarks of innate-like T-cells. While conventional CD8 T-cells are quite well characterized, CD8 T-cells sharing features of NK cells and memory CD8 T-cells, are a newly described immune cell population. They can be distinguished from conventional CD8 T-cells by their combined expression of panKIR/NKG2A and Eomesodermin (E), a unique phenotype closely associated with IFN-γ production in response to innate stimulation. Here, we first provided new evidence in favor of the innate character of panKIR/NKG2A(+) E(+) CD8 T-cells in normal subjects, documenting their position at an intermediate level in the innateness gradient in terms of both innate IFN-γ production and diminished mitochondrial mass. We also revealed that CD8 E(+) panKIR/NKG2A(+) T-cells, hereafter referred to as Innate E(+) CD8 T-cells, exhibit increased senescent (CD27(-) CD28(-)) phenotype, compared to their conventional memory counterparts. Surprisingly, this phenomenon was not dependent on age. Given that inflammation related to chronic viral infection is known to induce NK-like marker expression and a senescence phenotype among CD8 T-cells, we hypothesized that innate E(+) CD8 T-cells will be preferentially associated with exacerbated cellular senescence in response to chronic alloantigen exposure or CMV infection. Accordingly, in a pilot cohort of stable kidney allotransplant recipients, we observed an increased frequency of the Innate E(+) CD8 T-cell subset, together with an exacerbated senescent phenotype. Importantly, this phenotype cannot be explained by age alone, in clear contrast to their conventional memory counterparts. The senescent phenotype in CD8 T-cells was further increased in cytomegalovirus (CMV) positive serology transplant recipients, suggesting that transplantation and CMV, rather than aging by itself, may promote an exacerbated senescent phenotype of innate CD8 T-cells. In conclusion, we proposed that kidney transplantation, *via* the setting of inflammatory *stimuli* of alloantigen exposure and CMV infection, may exogenously age the CD8 T-cell compartment, especially its innate component. The physiopathological consequences of this change in the immune system remain to be elucidated.

## Introduction

### Human Innate CD8 T-Cells: Phenotypic and Functional Definition

During the 2000s, a population of T-cell receptor (TCR)αβ(+) CD8(+) thymocytes with a mature phenotype was described in mice (1–10). They expressed phenotypical markers (CD44(+) CD62L(-)) of immunological memory and produced IFN-γ in response to innate stimulation by the combination of cytokines IL-12 and IL-18 (8), while their differentiation depended on the transcription factor Eomesodermin (Eomes). This new pool of TCRαβ(+) CD8(+) cells has been referred to as innate-memory (IM) CD8 T-cells because of their antigen-inexperienced memory-phenotype together with the property of responding to innate stimuli. On a parallel track, a counterpart of this unique thymic TCRαβ(+) CD8(+) IM cell population, called “virtual memory (VM)” CD8 T-cells, has been described in peripheral lymphoid organs, particularly the spleen. As in IM CD8 T-cells, the differentiation of VM CD8 T-cells depends on Eomes. Moreover, an entire set of arguments in unimmunized mice has shown that this peripheral population possesses characteristics of antigen-inexperienced cells, phenotypic and functional features of memory cells (11), as well as rapid IFN-γ production after exposure to proinflammatory cytokines (11). The frequency of the VM CD8 T-cell population has been shown to increase with age while retaining effector anti-infectious functions (12–14).

At the same time, in humans, numerous studies have identified a population of TCRαβ(+) CD8(+) lymphocytes expressing NK receptors (NKR), such as KIR and NKG2A/C (15–19). Interestingly, these cells have a preferentially EMRA (CD45RA(+) CCR7(-) CD57(+)) phenotype along with weaker TCR signalling than that observed for the CD8(+) KIR/NKG2A/C(-) population in terms of IFN-γ and TNF-α expression and degranulation process (20). However, human TCRαβ(+) CD56(+) T-cells (enriched in KIR(+) cells) have been characterized by their unique propensity among the CD8 T-cell pool to produce IFN-γ in response to innate stimulation by the combination of IL-12 and IL-18 (21). The physiopathologic role of CD8(+) KIR/NKG2A(+) T-cells has remained unclear. They have been associated with loss of immune response efficiency during cancer, and also with greater immune response efficiency in some infection conditions. Indeed, Lies Boelen et al. showed that CD8(+) KIR(+) T-cells were preferentially present in “Elite Controller” patients during human immunodeficiency virus (HIV) infection. In this work, the authors provided arguments suggesting that the expression of NKR specific to self-MHC molecules plays a role in protecting the effector functions of HIV-specific CD8 T-cells (22).

**Figure 1:**
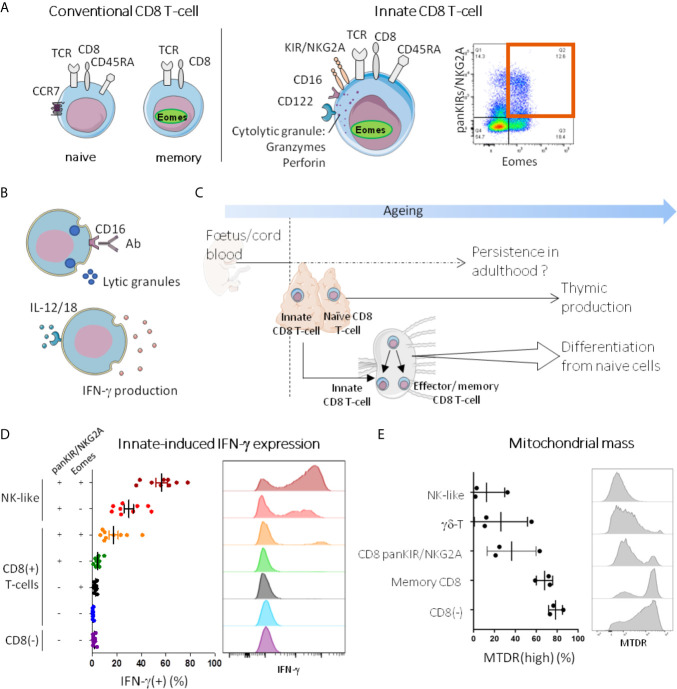
Innate T-cells in humans: current state of knowledge. **(A, B)** Innate T-cells are memory phenotype CD8 T-cells with innate functions. In humans, as their conventional memory counterparts (panel **A**, left), innate CD8 T-cells (panel **A**, middle) express TCRαβ and CD8, and have memory characteristics, as attested notably by Eomes expression and EMRA phenotype (CD45RA(+) CCR7(-)). They also share with NK cells some innate immune receptors such as KIR (KIR2D and KIR3DL1/2), NKG2A, CD122 and CD16. Co-expression of Eomes and KIR/NKG2A brings together the major fraction of CD8 T-cells able to free lytic granules in response to CD16 stimulation with antibodies (ADCC, antibody-dependent cellular cytotoxicity) and to produce IFN-γ upon innate-like stimulation by IL-12*/*IL-18 [(24) panel **B**]. As a result, CD8 T-cells co-expressing Eomes and KIR/NKG2A are identified as innate CD8 T-cells. A representative dot plot graph of Eomes(+) KIR/NKG2A(+) cells among CD8(+)CD3(+) PBMC from a healthy adult analyzed by flow cytometry is shown in panel **A** (right). **(C)** Ontogeny of innate CD8 T-cells. Innate CD8 T-cells have been evidenced in human cord blood, and like other fetal immune cells, they may be able to persist in adulthood. In mice, it is well-documented that they are produced in the thymus not only during the first stages of life but throughout a lifetime. With ageing, naive cells from the periphery differentiate into T-cell expressing innate features without antigen-stimulation. **(D, E)** Innate CD8 T-cells are part of the innateness gradient. PBMC from healthy donors were stained after 24h stimulation *in vitro* with IL-12/18 **(D)** or *ex vivo* **(E)**, and analyzed by flow cytometry for innate-induced IFN-γ expression and mitochondrial mass (MTDR), respectively. Each point represents one healthy adult donor. A representative histogram is shown for each cell population. For detailed gating strategy, see **Figure S1**.

In addition, several studies have shown that a large majority of KIR/NKG2A(+) CD8 T-cells express Eomes (23–26) and that Eomes expression is essential for innate functions such as IFN-γ expression after innate stimulation by IL-12/IL-18 or degranulation after CD16 stimulation (24, 25) (**Figures 1A, B**). Interestingly, IFN-γ-expressing KIR/NKG2A(+) Eomes(+) CD8 T-cells in response to IL-12/IL-18 have been found in fetal thymus/cord blood in humans (23–25), attesting to their education by a foreign antigen-independent differentiation. In addition, their enrichment in EMRA cells indicates that the acquisition of this phenotype is accompanied (from the selection phase) by characteristics of terminal differentiation (24).

This population has also been found in non-lymphoid tissues, cancerous lesions (27) and in normal liver with a threefold higher frequency than observed among peripheral blood mononuclear cells (PBMC) (25).

A positive correlation between the frequency of splenic KIR(+) Eomes(+) CD8 T-cells and subject age has been described, suggesting that at least part of this cell population is differentiated or accumulates with age (25). This population was called “VM” by White et al. (25) while we have used the expression “innate CD8 T-cells” (24, 27). On the basis of co-expression of KIR and NKG2A among EMRA cells, Quinn et al. identified a cell population that they also call “VM” (28, 29). The authors showed a positive correlation between frequency of this blood VM CD8 T-cell population and subject age.

In summary, in humans there exists an efflux of peripheral innate CD8 T-cells that appear very early in life and are likely to be maintained throughout life and, in addition, a probable contingent of innate CD8 T-cells appearing during a lifetime (**Figure 1C**).

Remarkably, in adulthood the CD8 T-cells generated during the neonatal period in mice maintain a greater potential response to the antigen or to a stimulation by IL-12/IL-18 (30). This observation is in line with the general and attractive hypothesis that, in humans and mice, fetal or neonatal T-cells could have more marked innate characteristics than adult T-cells (31). Specifically, these cells are able to produce IL-8 (32, 33), antimicrobial peptides (34), or have a propensity to respond more effectively to stimulation by IL-12/IL-18 (30, 31, 34).

**Figure 1D** illustrates data obtained from PBMC of healthy subjects showing the decisive role of Eomes in the acquisition of the IFN-γ response to stimulation by IL-12/IL-18 among panKIR/NKG2A-expressing CD8 T-cells. Indeed, on close examination, we noted the quasi-absence of IFN-γ expression by panKIR/NKG2A(+) Eomes(-) CD8 T-cells. This figure further demonstrates that the innate-dependent IFN-γ expression of panKIR/NKG2A(+) Eomes(+) CD8 T-cells is positioned with an intermediate profile between the two extremes of the spectrum ranging from “conventional” adaptive T-cells (CD4 T-cells) to NK cells (i.e. the archetype of the innate lymphoid cells, which also include “ILC”). The fact that in the control NK-like cell population, the proportion of IFN-γ expressing cells was 2-fold increased among Eomes-expressing cells, confirms the role of Eomes in the acquisition of innate IFN-γ expression.

Akbar et al. provided convincing arguments suggesting that EMRA CD8 T-cells expressed a metabolic reprogramming signature, evidenced by a decrease in mitochondrial biomass, resulting from decreased glucose and fatty acid absorption (35). The same group (35, 36) as well as Quinn et al. (28, 29), highlighted the senescent character of VM CD8 T-cells expressing NK markers (KIR and/or NKG2A), and thereby raised the question of a possible convergence between the senescent phenotype and the acquisition of an innate phenotype.

From this standpoint, it is interesting to note that the metabolic activity of resting NK cells is weakly oxidative (37–39). That is why we analyzed the mitochondrial biomass of innate CD8 T-cells (**Figure 1E**) and compared it to that of NK cells, at one end of the gradient, and adaptive CD4 T-cells, at the other end. We included γδ-T cells as a hallmark example of innate T-cells. Interestingly, the mitochondrial biomass of innate CD8 T-cells defined by the expression of panKIR/NKG2A and CD45RA was found to be low, equivalent to that of NK cells and γδ-T cells (**Figure 1E** and data not shown). So it is that in addition to innate-dependent IFN-γ production, metabolic status is another element visualizing the existence of a phenotypic gradient ranging from adaptive lymphocytes to innate lymphocytes with panKIR/NKG2A(+) Eomes(+) CD8 T-cells in between.

### The Innateness Concept

Hayday and his colleagues were pioneers in the description of innate functions carried out by T-cell populations and in their proposal to conceptualize/generalize this phenomenon (40). Previous works had suggested that unconventional T-cells, such as invariant natural killer T (iNKT) cells, have innate functions, including cytotoxicity independent of TCR engagement, IFN-γ expression in response to stimulation by IL-12/IL-18 or IL-12/IL-33 but without associating these functions with a significant decrease in TCR signalling (41–44).

**Figure 2:**
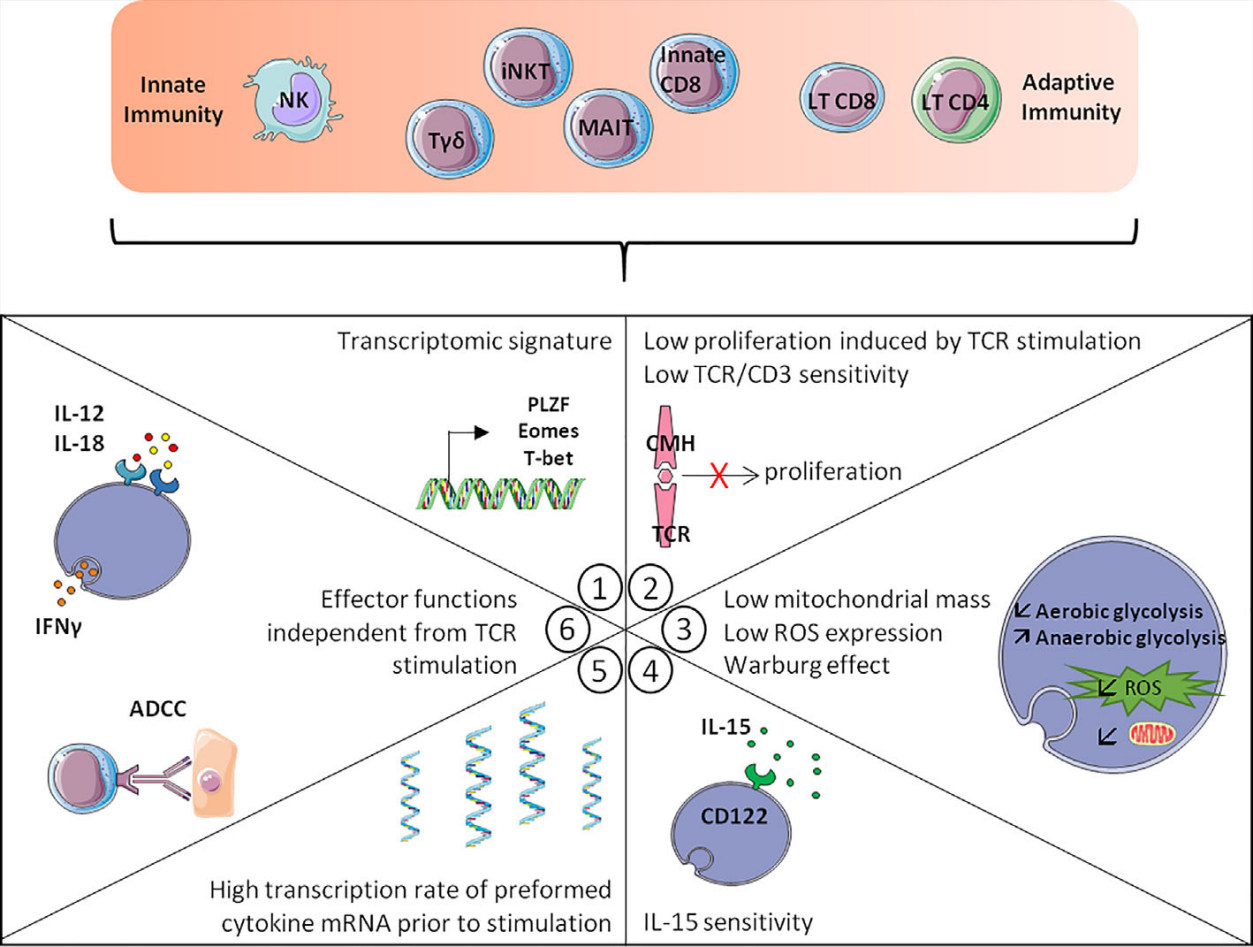
Hallmarks of innateness gradient. The more T-cells are close to innate cells like the NK-cells, the more they display the following hallmarks: 1/Transcriptomic signature with transcription factors expression such as PLZF, Eomes or T-bet. 2/Low TCR/CD3 sensitivity and low proliferation induced by TCR stimulation. 3/Metabolic changes with lower mitochondrial mass, lower ROS production and the Warburg effect. 4/IL-15 sensitivity, with increased CD122 expression. 5/A higher transcription rate of preformed cytokine mRNA prior to stimulation. 6/Effector functions independent from TCR stimulation such as ADCC and IFN-γ production after IL-12/IL-18 stimulation. Adapted from Gutierrez-Arcelys et al. (45).

Brenner et al. (45), using an RNA-seq analysis, described the existence of a phenotypic and functional signature common to innate lymphoid cells (NK cells and some ILC) and innate T-cells (MAIT, iNKT cells and γδ-T cells). This approach made it possible to conceptualize and globalize the notion of “innate stimulation”, which is based on the expression of a pool of transcription factors associated with the character of innateness, a profile of effector functions independent of TCR signalling, a high level of preformed transcripts encoding cytokines including IFN-γ, a weaker proliferative response to TCR stimulation than “conventional” T-cells, greater sensitivity to IL-15, particularly in the maintenance of homeostatic proliferation, and lastly, a “weakly oxidative” metabolic profile (**Figure 2**). Remarkably, a fraction of CD8 T-cells possess criteria bringing them closer to NK cells and innate T lymphocytes. Among the criteria of the innateness profile defined by RNA-seq analysis, KIR and NKG2 receptors and the transcription factors T-bet, HOXP, ZEB2 and PLZF are the most strongly associated with innateness, while Eomes is enriched in the innate compartment but at a lower level. When considering functional criteria, the most characteristic is IFN-γ response to IL-12/IL-18 stimulation and the sensitivity in this system to the action of IFN-γ which makes it possible to visualize the gradient of innateness from NK cells to CD8 T-cells (**Figure 1D**) (45). Another remarkable point is the relative independence of innate T-cells from TCR engagement, particularly with regard to proliferation (45), as illustrated by the dispensable character of TCR in the maintenance and survival of iNKT lymphocytes. Indeed, at steady-state, the level of Nur77 expression (a marker of TCR engagement) was lower in splenic iNKT cells than in conventional CD4 T-cells (46) while iNKT cells maintained functions and survival after *in vivo* ablation of their TCR (47). Another unique feature of innate T-cells is their sensitivity to IL-15, which is explained by increased expression of CD122, the IL-2/IL-15 receptor β chain (45), allowing IL-15 to maintain homeostatic expansion of iNKT cells (48–50) along with stable T-bet expression (49).

A final element contributing to the definition of innateness is metabolic status, more precisely, a low mitochondrial biomass (51, 52). At steady-state, the decreased mitochondrial biomass (51, 52) found in innate lymphocytes as compared to adaptive lymphocytes is correlated to lower glucose uptake (51) as well as lower reactive oxygen species production (45). At this stage, it seems important to consider that within the gradient of innateness, each of the “classic” populations can be sub-distributed on the basis of an individual signature, thereby demonstrating that within innate cells, a metabolic shift can be associated with the acquisition of adaptive traits. Indeed, human CD57(bright) NK cells, which partially consist in adaptive memory cells, display a higher mitochondrial biomass and more intense oxidative metabolism than their CD57(dim) counterparts (37, 53). Likewise, type 1 iNKT (iNKT1) cells, which preferentially express IFN-γ after TCR or IL-12/IL-18 stimulation, and whose phenotype closely depends on T-bet expression, exhibit a low mitochondrial biomass, in contrast to iNKT2 or iNKT3 cells, which present a higher mitochondrial mass and glucose uptake along with more intense oxidative metabolism (51, 54–56). With regard to these data, metabolic conversion of iNKT cells depends on mTOR (Mammalian Target of rapamycin) recruitment (51, 54–57).

### Phenotypic and Functional Characteristics of Senescent T-Cells

Age-related changes in immune responses make older people vulnerable to new infections or pathogens or may facilitate reactivation of latent bacterial or viral pathogens. The term used to describe this phenomenon is immunosenescence or senescence of the immune system. One of the hallmarks of immunosenescence is the gradual decrease of the naive lymphocyte cell pool, with a loss of potential/capacity for self-renewal.

**Figure 3:**
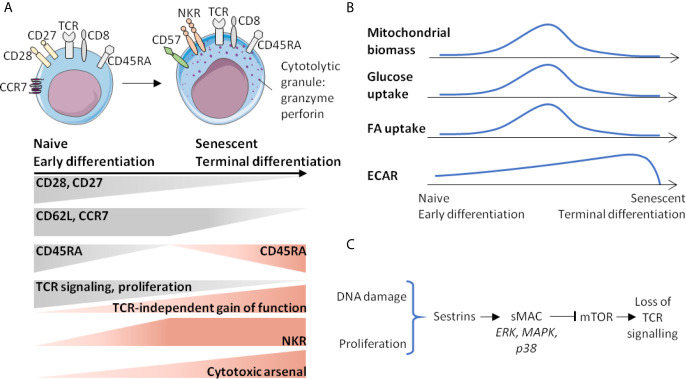
Senescence of CD8 T-cells is associated with phenotypic and metabolic changes. **(A)** Gradient of activation or function marker expression from naive (early differentiated) to senescent (terminal differentiated) cells. **(B)** Age-related metabolic changes of CD8 T-cells. **(C)** Events and signalling factors leading to the loss of TCR sensitivity that accumulate with age. ECAR, extracellular acidification rate; FA, fatty acid; NKR, NK receptors; sMAC, Sesn-MAPK activation Complex.

In humans, CD8 T-cells are generally considered to be the Achilles’ heel of immune homeostasis (58), due to the early and significant erosion of their naive CD8 T-cell component with age and accumulation/replacement by terminally differentiating/VM CD8 T-cells (28, 29). This phenomenon is accompanied by contraction of the TCR repertoire. The CD8 T-cell compartment exhibits approximately 10^8^ different TCR in (young) adult humans, while this diversity decreases by 3-to-5- fold in the elderly (59), it remains sufficient to respond to new antigens (58, 59). However, in situations of chronic stimulation of the immune system, including autoimmune or inflammatory diseases or infection by cytomegalovirus (CMV), erosion of CD8 T-cell repertoire diversity is more rapid (60–63). Age-related diversity erosion of the TCR repertoire is particularly marked in terminal differentiating memory (EMRA) CD8 T-cells, but it is also observable, albeit at a lower level, in the pools of effector-memory (EM) and naive CD8 T-cells (59). In humans, several profiles or markers are associated with an immunosenescence phenotype of CD8 T-cells: accumulation of EMRA phenotype cells, CD57 and NK receptor expression such as KIR, NKG2A/C and NKG2D (**Figure 3A**) (36, 58).

Senescent CD8 T-cells maintain a level of homeostatic proliferation that depends on IL-15 at the expense of IL-7, which is also a key cytokine controlling CD8 T-cell homeostatic proliferation. Indeed, age-related loss of sensitivity to IL-7, caused by decreased accessibility of the IL-7R gene to transcription should be taken into account (64). It is as if a shift were taking place in favor of a privileged proliferation of more differentiated CD8 T-cells expressing a higher level of CD122 (29). In addition, CD122 expression is increased by CD8 T-cells in elderly compared to young subjects (29). Finally, a decreased proliferative response to TCR stimulation, in addition to decreased TCR signalling, has also been proposed as a possible mechanism for differentiation/senescence programming, which would lead to an innate-type effector function gain (36). Another marker associated with terminal differentiation is a complex cytotoxic arsenal, in which perforin and granzymes (Gz) are highly expressed (36). A last phenotypic criterion associated with both terminal differentiation and immunosenescence of CD8 T-cells is the loss of CD27 and CD28 expression (**Figure 3A**) (36).

At steady-state, the mitochondrial biomass of EMRA CD8 T-cells is lower than that of effector memory (EM) CD8 T-cells and equivalent to that of naïve CD8 T-cells (65). The maximal mitochondrial respiration (by measuring OCR (Oxygen-Consumption-Rate) under decoupling conditions) is similar in EMRA and naive CD8 T-cells and lower than in EM CD8 T-cells (65). However, the part of mitochondrial metabolism devoted to ATP production is higher in the EMRA CD8 T-cell compartment, with higher amounts of preformed ATP (65). Moreover, EMRA CD8 T-cells also express many genes either associated with metabolic functions, including glucose uptake (Glut1), fatty acid uptake (carnitine palmitoyl transferase, CPT1A), lactic acid transport (SLC16A3) or which are involved in the pentose pathway (glucose-6-phosphate deshydrogenase, G6PD) (65). Finally, comparison of VM CD8 T-cells, defined by KIR/NKG2A expression and EMRA phenotype, in young or elderly subjects has shown that the VM CD8 T-cells of elderly subjects present superior maximal OCR, indicating active mitochondrial metabolism (29) (**Figure 3B**). All of these elements suggest a very active metabolism of EMRA CD8 T-cells, likely associated with increased IL-15 sensitivity (29, 65). Moreover, it can be assumed that senescent T-cells bring together a set of metabolic modifications distinct from those described in metabolic exhaustion during cancers or chronic infections (66, 67), thereby attesting to a specific senescence signature of senescent or terminal differentiation CD8 T-cells.

Sestrins (Sesn), which are evolutionarily-conserved stress-inducible proteins, have brought new knowledge about the molecular mechanisms involved in senescence and/or EMRA phenotype acquisition. They counteract oxidative stress, regulate mTORC1 (mTOR Complex 1) functions by mechanisms involving AMPK (an AMP-activated protein kinase) and Rag GTPases (68), and have anti-aging functions (68). Interestingly, Sesn expression is increased among senescent CD8 T-cells defined on the basis of CD27(-) CD28(-) phenotype (69). In these cells, Sesn interfere with p38, ERK, JNK and MAPKs (Mitogen-activated protein kinases), which will form a Sesn-MAPK activation Complex (sMAC) associated with cell survival, mTOR blocking and TCR signalling decrease (**Figure 3C**). Indeed, it has recently been suggested that Sesn2, one on the three Sesn family members, induces NKG2D expression and NK-like functions in senescent-like CD8 T-cells (70). In other words, senescent-like CD8 T-cells are not defective cells in end of terminal differentiation, but actively reprogrammed cells, raising the question of the role of Sesn2 in programming innate functions. Is Sesn2 expression and/or action restricted to senescent or terminally differentiating CD8 T-cells or also involved in innateness acquisition? Some of the response is provided by the public data associated with the work of Gutierrez-Arcelus et al. (45), which reveals that Sesn2 is significantly associated with innateness gradient (Beta 0.27; p <10-13).

One consequence of senescence is a low-grade systemic inflammation, also defined as inflammaging, with a chronic production of cytokines and pro-inflammatory molecules, such as TNF-α, IL-6, IL-1 or C-Reactive-Protein leading to a pro-inflammatory state which is predictive of death (71). This profile, called Senescence-Associated-Secretory-Phenotype (SASP) (71), is similar to that observed from senescent fibroblasts (63, 71) and should be considered as a warning signal for the immune system. In the elderly, T-cells, particularly CD8 T-cells, contribute to the production of cytokines involved in SASP. Different elements produced by CD8 T-cells are likely to be involved in the genesis of inflammation: TNF-α, cytokines of the IL-1 family, as well as molecules belonging to the cytotoxic arsenal (72). GzB is one of the factors likely to be involved in inflammation, through its activity on molecules of the extracellular matrix (Extracellular molecules) and on the activation of IL-1 or IL-18 (72). Another element of the cytotoxic arsenal likely involved in SASP is GzK, which is highly expressed by a population of CD8 T lymphocytes, TOX(+) Eomes(+) T-bet(low) PD-1(+) (73). GzK has the particularity of being able to induce SASP production *in vivo* (73, 74).

It has been reported that chronic stimulation can induce senescence together with NKR expression in CD8 T-cells in several pathologic situations, such as viral infection (75), chronic inflammatory disease (76) or cancer (77). Considering that allo-transplantation is also a condition of chronic stimulation, i.e., in response to continued exposure to allo-antigens, we then analyzed the senescence/NKR-expression status of the CD8 T-cell pool, with particular attention given to its innate contingent, in long-term stable kidney transplant patients (mean 20 years) treated with cyclosporine A (CsA) monotherapy. To this aim, we first investigated in normal subjects, among CD8 T-cells, whether there is or not a particular association between innate (panKIR/NKG2A(+) Eomes(+)) CD8 T-cells and senescence and/or age. Then, we explore the phenotype of conventional and innate CD8 T-cells in allotransplant patients, particularly those associated with positive cytomegalovirus (CMV) serology.

## Materials and Methods

### PBMC

The study protocol was approved by the ethics committee at Poitiers University Hospital (Ethics Committee Ouest III, Poitiers, France, registration number 16.10.42), and the study was conducted in compliance with Good Clinical Practice guidelines. All participants gave written informed consent before inclusion in the trial. This clinical trial was registered in the international clinical trial registry ClinicalTrials.gov, Identifier: NCT03227965. PBMC were isolated from blood samples by density gradient centrifugation (Histopaque^®^-1077, Sigma-Aldrich, St Louis, MO, US), resuspended in 90% Fetal Bovine Serum (FBS) (10270106, Gibco^®^, Thermo Fisher Scientific, Waltham, MA, US) with 10% DMSO (D2650, Sigma-Aldrich, St Louis, MO, US), and frozen at -80°C and then transferred in liquid nitrogen until use.

Frozen PBMC from 23 healthy donors (HD) (median age 46 years, range: 21363, sex ratio: 11/12) were obtained from the French Blood Institute (EFS, Lyon, France).

See **Table 1** for cohort description. Briefly, inclusion criteria are: delay of kidney transplantation >10 years, stable renal function, treatment with cyclosporine A (CsA) monotherapy.

**Table 1:** Patient and healthy donor characteristics.

	AlloTx	HD
Participants (n)	46	23
Sex		
Women (n)	15	11
Men (n)	31	12
Age (yr)	66 (46-85)	46 (22-63)
Time after transplantation (yr)	18 (10-29)	/
Cancer (n)	14	/
CsA trough level (ng/ml)	93 (67-129)	/

HD, healthy donors; AlloTx, kidney-transplant patients.

### Cell Culture

For *ex vivo* pro-inflammatory stimulation, after thawing, PBMC from patients and HD were cultured (1x10^6^ cells/mL) 24 well plate in RPMI 1640 medium supplemented with 10% heat-inactivated FBS and antibiotics with IL-12 and IL-18 for 2 days (20 ng/mL of each cytokine, R&D Systems (Minneapolis, MN, US) and MBL International (Woburn, MA, US) respectively). For assessment of IFN-γ secretion, Golgistop (BD 554724, Becton, Dickinson & Compagny, Franklin Lake, NJ, US) was added for the last 5 h of culture.

### Flow Cytometry

Phenotypic analysis of PBMCs was performed by flow cytometry either *ex vivo* or after cell culture. Expression of different markers was assessed by staining with appropriate combinations of antibodies. A detailed list of the antibodies used to stain cells is provided in **Supplementary Table**. panKIR/NKG2A referred to staining with a mixture of the three following antibodies from Miltenyi Biotec (Bergisch Gladbach, Germany) KIR2D, KIR3DL1/KIR3DL2 (CD158e/k) and NKG2A (CD159a). Briefly, dead cells were excluded by using the Zombie NIR™ Fixable Viability Kit (BioLegend) stained for membranous markers and fixed permeabilized with the FoxP3/Transcription Factor Staining kit (eBioscience^TM^) before intranuclear staining. Flow data were acquired on a FACsVerse flow cytometer (Becton, Dickinson & Compagny) with FACSuite™ software (Becton, Dickinson & Compagny) and analyzed using FlowJo™ v10 (Becton, Dickinson & Compagny).

### Statistical Analysis

Data are expressed in mean +/- SEM. The statistical significance of differences in mean values was analyzed by the Mann–Whitney or Wilcoxon non-parametric test. The Pearson correlation test was used to test the association between cell frequencies and age. Results were considered to be statistically significant when p<0.05. *p<0.05; **p<0.01; ***p<0.001, ns not significant. Statistical analysis was performed using GraphPad Prism version 7.0 (GraphPad Software). Significant outliers were identified using the Grubbs’ test and excluded from analysis.

## Results

### PanKIR/NKG2A(+) Eomes(+) and panKIR/NKG2A(+) Eomes(-) CD8 T-Cells Share Innate Functions and a Senescent/Inflammaging-Like Signature

**Figure 4:**
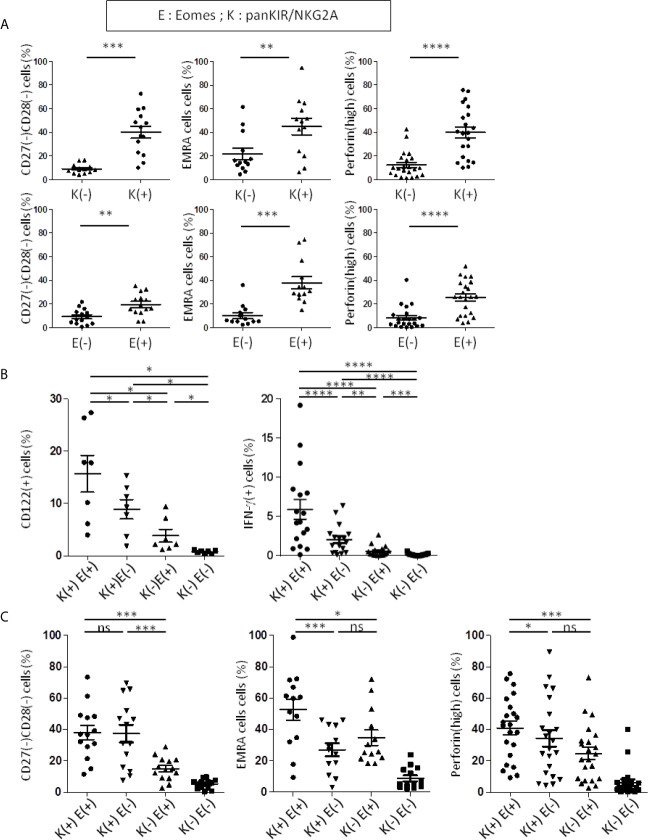
PanKIR/NKG2A(+) Eomes(+) and panKIR/NKG2A(+) Eomes(-) CD8 T-cells share innate functions and a senescent/inflammaging-like signature. Flow cytometry analysis of TCRαβ(+) CD8(+) circulating live lymphocytes from healthy donors. **(A)** CD8 T-cells expressing panKIR/NKG2A or Eomes markers preferentially harbor a senescent signature and a terminal effector phenotype. The frequencies of senescent (CD27(-) CD28(-)) (n=14), EMRA (CD45RA(+) CCR7(-)) (n=13) and perforin(high)-expressing cells (n=22) among CD8 T-cells expressing panKIR/NKG2A (upper panel) or Eomes (lower panel) were analyzed. **(B)** panKIR/NKG2A and Eomes markers define two innate CD8 T-cell subsets besides their conventional memory and naive counterparts: evidence for an innateness gradient. CD122-expressing cells (n=7) *ex vivo* (left panel) and IFN-γ-producing cells (n=17) (right panel) frequencies after 48h stimulation with IL-12/IL-18 among the four CD8 T-cell subpopulations defined by panKIR/NKG2A and Eomes markers. Representative plots are shown in **Figure S2**. **(C)** Innate CD8 T-cell subsets are enriched in senescent/terminal effector cells. Frequencies of senescent (CD27(-)CD28(-)) cells (n=14) (left panel), EMRA cells (n=13) (middle panel) and perforin(high)-expressing cells (n=22) (right panel) among the four CD8 T-cell subpopulations defined by panKIR/NKG2A and Eomes markers. Differences between E(-)K(-) and the other three cell populations, although significant, are not shown. Data are presented as mean ± SEM. Two-tailed Wilcoxon non-parametric test. *p < 0.05; **p < 0.01; ***p < 0.001; ****p < 0.0001; ns, not significant. For detailed gating strategy, see **Figure S1**.

From a cohort of healthy donors (HD), we showed that the expression of panKIR/NKG2A among TCRαβ(+) CD8(+) peripheral blood cells (for gating strategy, see **Figure S1**) is associated with senescence (CD27(-) CD28(-)) (**Figure S2A**), EMRA (CD45RA(+) CCR7(-)) (**Figure S1**) and inflammaging (based on our work on high expression of perforin, which is an element of the cytotoxic arsenal as well as a surrogate for granzymes involved in inflammaging; **Figure S2B**; **Figure 4A** top panels) phenotypes. Similarly, when considering expression of the transcription factor Eomes, enrichment of the same phenotypic criteria was observed, in a less pronounced but still significant manner (**Figure 4A** bottom panels).

From these data, we decided to systematically analyze the four CD8 T-cell compartments defined by the expression of panKIR/NKG2A and Eomes, our objective being to determine the respective contributions of these two factors to the acquisition of innate functions as well as EMRA, senescence and inflammaging signatures (**Figures 4B, C**). As expected, among the four CD8 T-cell compartments, panKIR/NKG2A(+) Eomes(+) CD8(+) T-cells, in accordance with their recently described innate-like functional features (24), displayed the most pronounced expression of CD122 as well as the unique property of producing IFN-γ in response to innate-like stimulation (IL-12/IL-18) (**Figure 4B**; **Figures S2C, D**) for this innate-like function. As for the cell compartment panKIR/NKG2A(+) with no expression of Eomes, it produces significantly smaller amounts of IFN-γ in the same experimental setting, thereby indicating that both NKR and Eomes are required for optimal achievement of this innate function. Quite differently, the signature of senescence (CD27(-) CD28(-)-expressing cells) was predominantly associated with innate (panKIR/NKG2A(+)) CD8 T-cells, regardless of whether or not Eomes was co-expressed (**Figure 4C** left panel). On the other hand, Eomes expression by panKIR/NKG2A(+) cells was required for the acquisition of EMRA phenotype (**Figure 4C** middle), given the fact that panKIR/NKG2A(+) Eomes(+) CD8(+) T-cells, hereinafter referred to as Innate E(+), are mostly EMRA (24) (**Figure S4** top left panel), while their Eomes(-) counterparts, hereinafter referred to as Innate E(-) cells (**Figure S4** top right panel), consist mainly of effector memory cells. As for perforin expression (**Figure 4C** right), a declining gradient was observed, starting from panKIR/NKG2A(+) cells with Eomes expression, then without Eomes expression and finally with Eomes-expressing cells harboring panKIR/NKG2A, suggesting that Eomes and NKR work as additional/independent factors for the acquisition of their cytotoxic arsenal, a feature that is also considered to be associated with inflammation, and possibly with age. The data from **Figure 4C** are conciliable with the conclusion that Innate E(+) CD8 T-cells co-associate senescence and inflammaging signatures. However, the innate phenotype of CD8 T-cells gathering Eomes and panKIR/NKG2A receptors is associated with an EMRA phenotype, while the senescent phenotype based on the loss of expression of CD27 and CD28 is associated with NKR expression. In addition, in the present report, we have identified a second innate-like subset E(-) compartment exhibiting intermediate/low CD122 expression and IL-12/IL-18-stimulated IFN-γ production, which is also enriched in senescent and inflammaging cells.

As mentioned above, a general consensus exists in the literature on the existence of an association between CD8 T-cell senescence and age (28, 29). However, when considering innate-CD8 T-cell populations individually, the studies are not concordant. In our report, Innate E(+) and Innate E(-) cell frequencies in HD do not appear to vary with age (**Figure S3**). In clear contrast, and as expected, panKIR/NKG2A(-) Eomes(+) CD8 T-cells, which primarily harbor an effector/memory phenotype, ((24); **Figure S4** bottom left panel), and that we hereinafter refer to as conventional Conv E(+) cells, are positively correlated with age, whereas their Conv E(-) counterpart, which primarily harbors a naive phenotype (**Figure S4** bottom right panel), as in a balance, is negatively correlated (**Figure S3**).

### Long-Term Allogeneic Kidney Transplantation Promotes the Generation of Innate CD8 T-Cell Subsets Together With Their Senescent/Inflammaging Signature

One element associated with the appearance of senescent immune cells and the appearance of innate CD8 T-cells in humans and modelling in mice is the effect of chronic immune/antigen stimulation (58, 63). From this standpoint, allogeneic kidney transplantation is a remarkable situation insofar as it results in stimulation by an allogeneic (and therefore foreign) antigen over several years or decades. In this context, we analyzed the senescent, terminal differentiation and inflammaging phenotypes of innate CD8 T-cells in a cohort of patients with more than 10 years of transplantation without rejection under CsA monotherapy (AlloTx group) (**Table 1**) (78).

**Figure 5:**
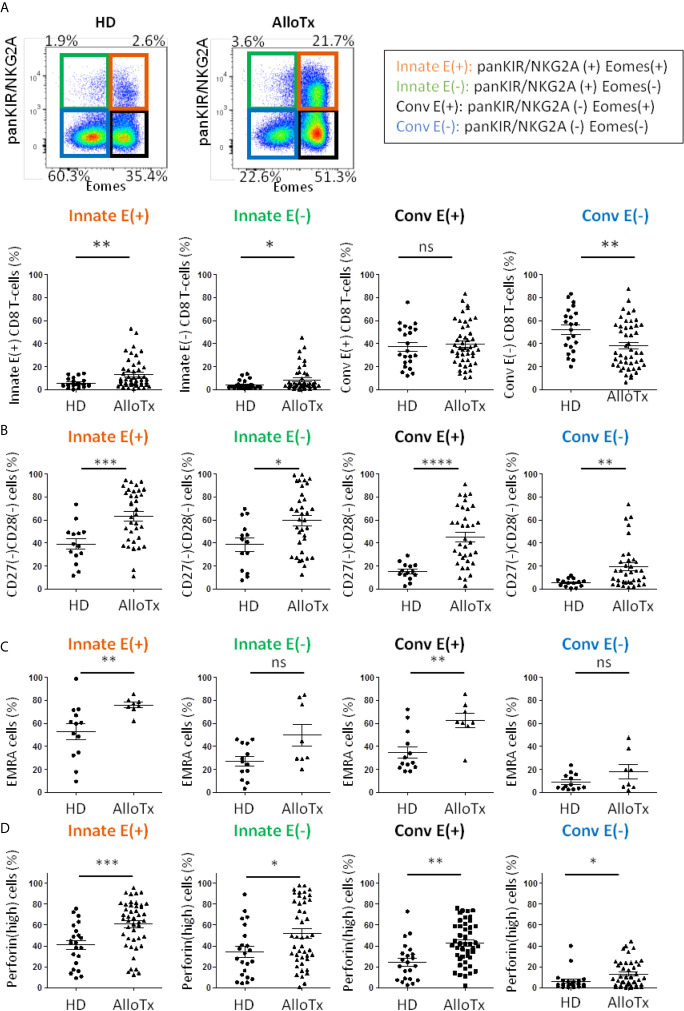
Chronic allo-antigenic stimulation promotes the generation of innate CD8 T-cell subsets and a senescent/inflammaging signature. **(A)** Frequencies of innate CD8 T-cell subpopulations are selectively increased in long-term kidney allograft recipients. Frequencies of CD8 T-cell subpopulations according to panKIR/NKG2A and/or Eomes expression among TCRαβ(+) CD8(+) live lymphocytes in healthy donors (HD, n=22) and long-term kidney allograft recipients (AlloTx, n=47). Representative plots are shown for one HD and one AlloTx recipient. **(B–D)** Senescent, EMRA and perforin(high) CD8 T-cell frequencies are increased in long-term kidney allograft recipients. Frequencies of senescent (CD27(-)CD28(-)) cells (HD n=15, AlloTx n=35) **(A)**, EMRA (CD45RA(+) CCR7(-); HD n=13, AlloTx n=8) **(B)** and perforin(high)- expressing cells (HD n=22, AlloTx n=45) **(C)** in CD8 T-cell subpopulations. In the Conv E(-) cell pool from AlloTx patients, the increased frequency of EMRA cells (**Figures 5** and **S4**) could explain the relatively high frequency of senescent cells (19% *vs* 12% in healthy controls). Representative plots are shown for one HD and one AlloTx recipient in **Figure S2**. Each dot represents one HD or AlloTx recipient. Two-tailed Mann-Whitney non-parametric test. *p < 0.05; **p < 0.01; ***p < 0.001; ****p < 0.0001; ns, not significant. For detailed gating strategy, see **Figure S1**.

Remarkably, in this cohort of transplant patients, as compared to HD, frequencies of the two Innate E(+) and E(-) CD8 T-cell compartments appeared to be significantly increased, while that of Conv E(+) CD8 T-cells remained unmodified. As a result, Conv E(-) CD8 T-cell frequency appeared to be substantially reduced (**Figure 5B** and **Figure S5**).

Moreover, markers of senescence (CD27(-) CD28(-)) (**Figure 5B**), terminal differentiation (EMRA phenotype) (**Figure 5C**) and inflammaging (perforin(high) expression) (**Figure 5D**) appeared to be exacerbated in innate CD8 T-cells, particularly in the Innate E(+) subpopulation of the AlloTx group. However, this phenomenon did not appear to be restricted to innate CD8 T-cell compartments, as since a similarly increased profile likewise occurred in the Conv E(+) CD8 T-cell compartment, thereby raising the question of a partial decoupling between acquisition of the innate-like phenotype and acquisition of a senescent inflammaging and/or EMRA phenotype.

Lastly, as is the case for HD, there is no correlation between frequency of innate CD8 T-cells and age, a finding once again contrasting with the significantly positive correlation frequently found with Conv E(+) CD8 T-cells (**Figure S5**). To definitively ensure that there is no age-related bias in our comparison between the HD and AlloTx groups, we matched the subjects of the two groups according to age. Even so, in AlloTx groups, the selective increase in the frequency of Innate E(+) and Innate E(-) CD8 T-cells remained nearly intact, as did the exacerbation of their senescent character (CD27(-) CD28(-)) and their perforin(high) expression, both of which appear to suggest inflammaging (**Figure S6**).

All of these data argue in favour of reprogramming CD8 T-cell populations, particularly its innate components independently of Eomes expression, under the influence of chronic stimulation linked to kidney transplantation rather than inherent to age.

### Chronic Stimulation by CMV in Renal Transplant Patients Is Associated With General Exacerbation of the Senescent/Inflammaging-Like Signature in the CD8 T-Cell Compartment

**Figure 6:**
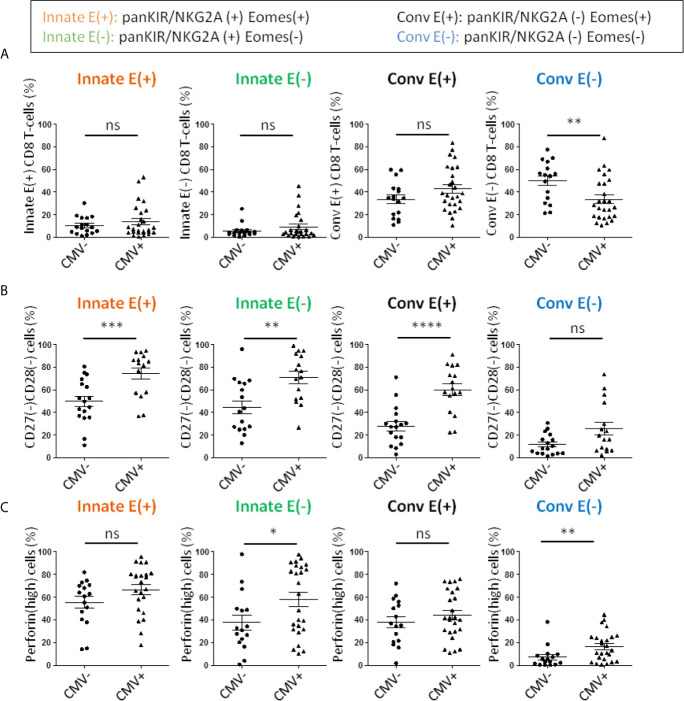
Frequencies of senescent and perforin(high) CD8 T-cells are increased in long-term kidney allograft with a CMV positive serology. **(A)** Frequencies of CD8 T-cell subpopulations according to panKIR/NKG2A and/or Eomes expression among TCRαβ(+) CD8(+) live lymphocytes in CMV(-) (n=17) or CMV(+) (n=26) long-term kidney allograft (AlloTx) recipients. **(B)** Senescent (CD27(-)CD28(-)) cells (CMV(-) n=17; CMV(+) n=16) and **(C)** perforin(high)-expressing cells (CMV(-) n=16; CMV(+) n=25) in CD8 T-cell subpopulations in AlloTx recipients. Two-tailed Mann-Whitney non-parametric test. *p < 0.05; **p < 0.01; ***p < 0.001; ****p < 0.0001; ns, not significant.

CMV infection is another event associated with accelerated ageing of the immune system, particularly the CD8 T-cell compartment. During kidney transplantation, CMV infection is associated with an increased risk of rejection and long-term complications of the transplant (79, 80). Interestingly, in our cohort, there was no difference in the frequency of Innate E(+) or E(-) CD8 T-cells or Conv E(+) cells, a finding suggesting that stimulation by CMV did not impact the generation of innate CD8 T-cells (**Figure 6A**). On the other hand, we observed a significant decrease in Conv E(-) CD8 T-cells, a compartment mainly represented by naive cells. This phenomenon could result from an increase in the global E(+) compartment (data not shown). All in all, among innate CD8 T-cell compartments, patients with positive CMV serology showed a further significant increase in frequency of senescent (CD27(-) CD28(-)) and perforin(high) expressing phenotypes (**Figures 6B, C**). However, while the exacerbated inflammaging signature (perforin(high)) associated with chronic CMV stimulation appears to selectively impact innate CD8 T-cells, this is not the case with senescence, which in fact impacts the four compartments defined by panKIR/NKG2A and Eomes expression.

On the whole, matters proceed as if chronic stimulation by allo-antigens and CMV possesses the quality of inducing/increasing a senescence/inflammaging signature in conventional CD8 T-cell populations and of exacerbating them in innate CD8 T-cell populations. In addition, events associated with the long-term status of the transplantation, including chronic stimulation by allo-antigens or even immunosuppressive treatment, could well be responsible for the increased numerical representation of innate CD8 T-cells.

The increased senescent phenotype is a global phenomenon in CD8 T-cells, which cannot be attributed to only one sub-population. From our results in AlloTx patients, we expected that, as is the case for cell frequencies, senescent and inflammaging phenotypes would be related to age in Conv T-cells, but not in innate CD8 T-cells. This assumption is confirmed by positive correlations between age of AlloTx patients and CD27(-) CD28(-) and perforin(high) frequencies among Conv CD8 T-cells (although not significant for perforin (p=0.08)), but not their Innate counterpart, as illustrated in **Figure S7**. As a result, Conv CD8 T-cells are affected by aging, as previously documented (58, 63), whereas innate CD8 T-cells are impacted by chronic stimulation rather than age.

## Discussion

It is well-documented that conventional CD8 T-cells are highly susceptible to senescence with chronologic age (58). This phenomenon is associated with a decrease in the naive CD8 T-cell pool together with accumulation of memory/terminally differentiated CD8 T-cells (28, 29).

Here, we postulated that organ transplantation is likely to alter the age-related senescence program of CD8 T-cells. Since some CD8 T-cells expressing innate markers have a propensity to display a senescent phenotype, we focused on innate (panKIR/NKG2A(+)) CD8 T-cells, which preferentially harbor a terminally differentiated phenotype.

We were thereby able to demonstrate for the first time that renal allotransplantation is accompanied by an increased frequency of Innate E(+) CD8 T-cells together with an exacerbated senescent phenotype (CD27 (–) CD28(-)), which is also apparent in the conventional CD8 T-cell pool, although to a lesser degree.

Since studies about T-cells in healthy subjects have shown that aging can trigger expression of NK-cell markers on CD8 T-cells (28, 70), we analyzed the influence of age in the generation and senescent phenotype of Innate E(+) CD8 T-cells in AlloTx recipients. Intriguingly, neither the generation nor the exacerbated senescent status of Innate E(+) CD8 T-cells could be explained by age alone, as evidenced by the significant differences that remained between patients and age-matched healthy controls. In addition, as expected, and in clear contrast with the Innate E(+) CD8 T-cell subset, its conventional memory counterpart was positively correlated with age in the same transplant recipients, in terms of both frequency and senescence. Lastly, we documented that this phenomenon preexists in healthy controls. Indeed, frequency of Innate E(+) CD8 T-cells was apparently not related to age, corroborating findings from Kasakovski et al. obtained with a larger healthy cohort aged from 6 to 84 years (81). Moreover, the healthy control Innate E(+) CD8 T-cell population appeared more enriched in senescent cells compared to its conventional memory counterpart, whose senescent phenotype increased with age.

All in all, these data suggest that in healthy controls, Innate E(+) CD8 T-cells acquire their senescent phenotype as part of their own differentiation program, and that in AlloTx patients, the increased frequency and exacerbated senescent phenotype of innate E(+) CD8 T-cells may be attributed to chronic allogeneic stimulation rather than to chronological age. The notion that T-cell senescence increases after kidney transplantation was previously evidenced by measuring telomerase activity, which was lower in transplant recipients five years after kidney transplantation (82). In the future, it would be interesting to compare AlloTx recipients and healthy subjects for telomerase activity in the CD8 T-cell compartment, and particularly its Innate E(+) contingent.

During the physiological aging that occurs in the elderly, a low grade of systemic inflammation is generally observed, referred to as inflammaging. Since it is well-recognized that chronic inflammation occurs from the first few months after kidney transplantation (83), we raised the possibility of an inflammaging phenotype (defined by perforin(high) expression) in the CD8 T-cell compartment from AlloTx recipients, and its conceivable association with senescence. Our observation of the increased frequency of perforin(high)-expressing cells in the four innate and conventional CD8 T-cell compartments in AlloTx patients led us to suggest that inflammaging occurs in AlloTx patients by impacting the whole CD8 T-cell compartment. However, senescence and inflammaging appeared at least partially non-interdependent in AlloTx patients. Indeed, when considering patients with positive CMV serology, even though a further increase of senescence frequency was found in all CD8 T-cell subsets as compared to patients with CMV negative serology, perforin(high), expressing cell frequency appeared increased in Innate E(-) CD8 T-cells but not in their Innate E(+) counterparts. These findings suggest that CMV and allogeneic chronic stimulations do not seem to have the same target cells and probably use different mechanisms, affecting Innate E(-) and E(+) CD8 T-cells differently. Further studies using a more direct marker of inflammaging, such as GzK (73, 74), are needed to definitively validate this assumption. Although CMV is one of the most widely studied viruses in transplant patients, other viruses may be implicated and should be analyzed, as infection is a major issue in transplantation, particularly as regards oncogenic viruses (84, 85).

An important issue that remains to be explored is the physiopathologic significance in AlloTx recipients of our observation of increased senescence and inflammaging among CD8 T-cells, particularly in Innate CD8 T-cells. We presume that this “age-like” phenotype is related to alterations in immune system competence. Considering innate CD8 T-cells, we categorized them as an NK-like CD8 T-cell subset that constitutively expresses CD122 and can rapidly produce IFN-γ on innate triggering (24) (**Figure 4B**), two elements of the innateness gradient described by Gutierrez-Arcelus et al. (45). In this respect, we confirmed *via* metabolic analysis that in the innateness gradient (45) innate CD8 T-cells represent an intermediate category between NK cells and adaptive CD4 T-cells. Further studies are needed to determine whether one or more elements of the innateness gradient (for details, see **Figure 2**) are lost in this unique CD8 T-cell subset in AlloTx recipients.

At the clinical level, one limitation of our pilot study is the absence of association between the CD8 T-cell subtypes described and clinical phenotypes of rejection, which is due to the fact that inclusion criteria of the AlloTx cohort were delay of kidney transplantation exceeding 10 years, and stable renal function. In spite of that, when considering a possible link between senescent Innate E(+) CD8 T-cells and graft rejection/tolerance, a recent study reported that higher frequency of EMRA CD8 T-cells at one year post-transplantation was associated with poor prognosis and graft failure (86). Given that Innate E(+) CD8 T-cells are enriched in the EMRA phenotype (24), and that this signature is exacerbated in AlloTx recipients, a prospective study monitoring innate CD8 T-cells with regard to clinical signs of rejection, infection or altered vaccine response, merits special consideration.

This study raises the possibility *in fine* that monitoring of innate CD8 T cells could represent an innovative approach for evaluation of transplant recipients, with major potential implications for management of immunosuppression. With this in mind, the data obtained in our pilot cohort should first be confirmed by extending our exploration in patients receiving immunosuppressive drugs other than CsA, especially tacrolimus, mycophenolic acid and glucocorticoids, which are frequently used in renal allotransplant recipients. Another limitation of our study is the small cohort size and young age of healthy controls.

In conclusion, we propose that kidney transplantation, *via* the setting of inflammatory stimuli of alloantigen exposure and CMV infection, may exogenously age the CD8 T-cell compartment, especially Innate E(+) CD8 T-cells. It would also be interesting to investigate whether this new hypothesis applies to other pathological situations of chronic immune stimulation such as autoimmunity or cancers.

## Data Availability Statement

The raw data supporting the conclusions of this article will be made available by the authors, without undue reservation.

## Ethics Statement

The studies involving human participants were reviewed and approved by Comité de protection des personnes Ouest III (assay number: ELITE, protocole number: 16.10.42, identification number: 2016-A01508-43). CHU de Poitiers, Pavillon administratif, entrée numéro 3 porte 213, 2 rue de la Milétrie, CS 90577, 86 021 Poitiers Cedex, France. The patients/participants provided their written informed consent to participate in this study.

## Author Contributions

LD designed the experiments, performed the experiments, analyzed and interpreted the data, and wrote the manuscript. MT and CL contributed to sample preparation from patients and healthy controls, designed the experiments, performed the experiments, and analyzed and interpreted the data. AT provided clinical samples and contributed to the interpretation of data. AB, AH, and J-MG together were responsible for the overall study design, supervised the project, and took primary responsibility for writing the manuscript. All authors contributed to the article and approved the submitted version.

## Funding

This study was supported by Ligue contre le Cancer du Grand Ouest (Comités départementaux de la Vienne, de la Charente, de la Charente Maritime) and Sport and Collection. This study was supported by INSERM, CHU de Poitiers, Université de Poitiers.

## Conflict of Interest

The authors declare that the research was conducted in the absence of any commercial or financial relationships that could be construed as a potential conflict of interest.
