# Supplementary material for: Allotransplantation Is Associated With Exacerbation of CD8 T-Cell Senescence: The Particular Place of the Innate CD8 T-Cell Component
Source: Front Immunol. 2021 Jul 21;12:674016. doi: 10.3389/fimmu.2021.674016 (PMC8334557; doi:10.3389/fimmu.2021.674016)
Supplement: Supplementary file 1 [file Presentation_1.pptx]

## Slide 1
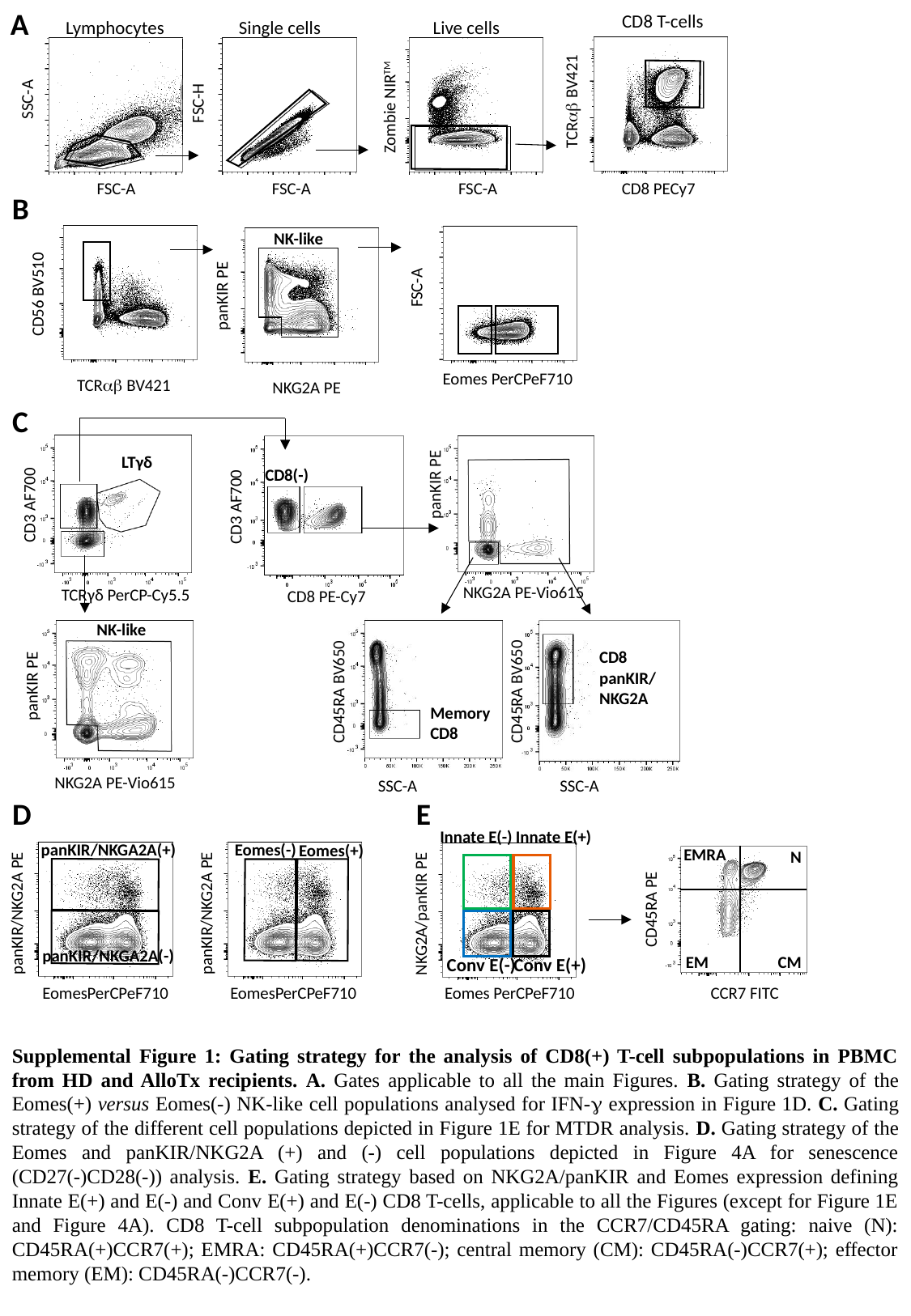

A
CD8 T-cells
TCRαβ BV421
CD8 PECy7
Lymphocytes
SSC-A
FSC-A
Single cells
FSC-H
FSC-A
Live cells
Zombie NIRTM
FSC-A
B
NK-like
FSC-A
CD56 BV510
panKIR PE
Eomes PerCPeF710
TCRαβ BV421
NKG2A PE
C
LTγδ
CD8(-)
panKIR PE
CD3 AF700
CD3 AF700
NKG2A PE-Vio615
TCRγδ PerCP-Cy5.5
CD8 PE-Cy7
NK-like
CD8 panKIR/NKG2A
panKIR PE
CD45RA BV650
CD45RA BV650
Memory CD8
NKG2A PE-Vio615
SSC-A
SSC-A
D
Eomes(-)
Eomes(+)
panKIR/NKG2A PE
EomesPerCPeF710
panKIR/NKGA2A(+)
panKIR/NKG2A PE
panKIR/NKGA2A(-)
EomesPerCPeF710
E
Innate E(-)
Innate E(+)
NKG2A/panKIR PE
Conv E(-)
Conv E(+)
Eomes PerCPeF710
EMRA
N
CD45RA PE
EM
CM
CCR7 FITC
Supplemental Figure 1: Gating strategy for the analysis of CD8(+) T-cell subpopulations in PBMC from HD and AlloTx recipients. A. Gates applicable to all the main Figures. B. Gating strategy of the Eomes(+) versus Eomes(-) NK-like cell populations analysed for IFN-γ expression in Figure 1D. C. Gating strategy of the different cell populations depicted in Figure 1E for MTDR analysis. D. Gating strategy of the Eomes and panKIR/NKG2A (+) and (-) cell populations depicted in Figure 4A for senescence (CD27(-)CD28(-)) analysis. E. Gating strategy based on NKG2A/panKIR and Eomes expression defining Innate E(+) and E(-) and Conv E(+) and E(-) CD8 T-cells, applicable to all the Figures (except for Figure 1E and Figure 4A). CD8 T-cell subpopulation denominations in the CCR7/CD45RA gating: naive (N): CD45RA(+)CCR7(+); EMRA: CD45RA(+)CCR7(-); central memory (CM): CD45RA(-)CCR7(+); effector memory (EM): CD45RA(-)CCR7(-).

## Slide 2
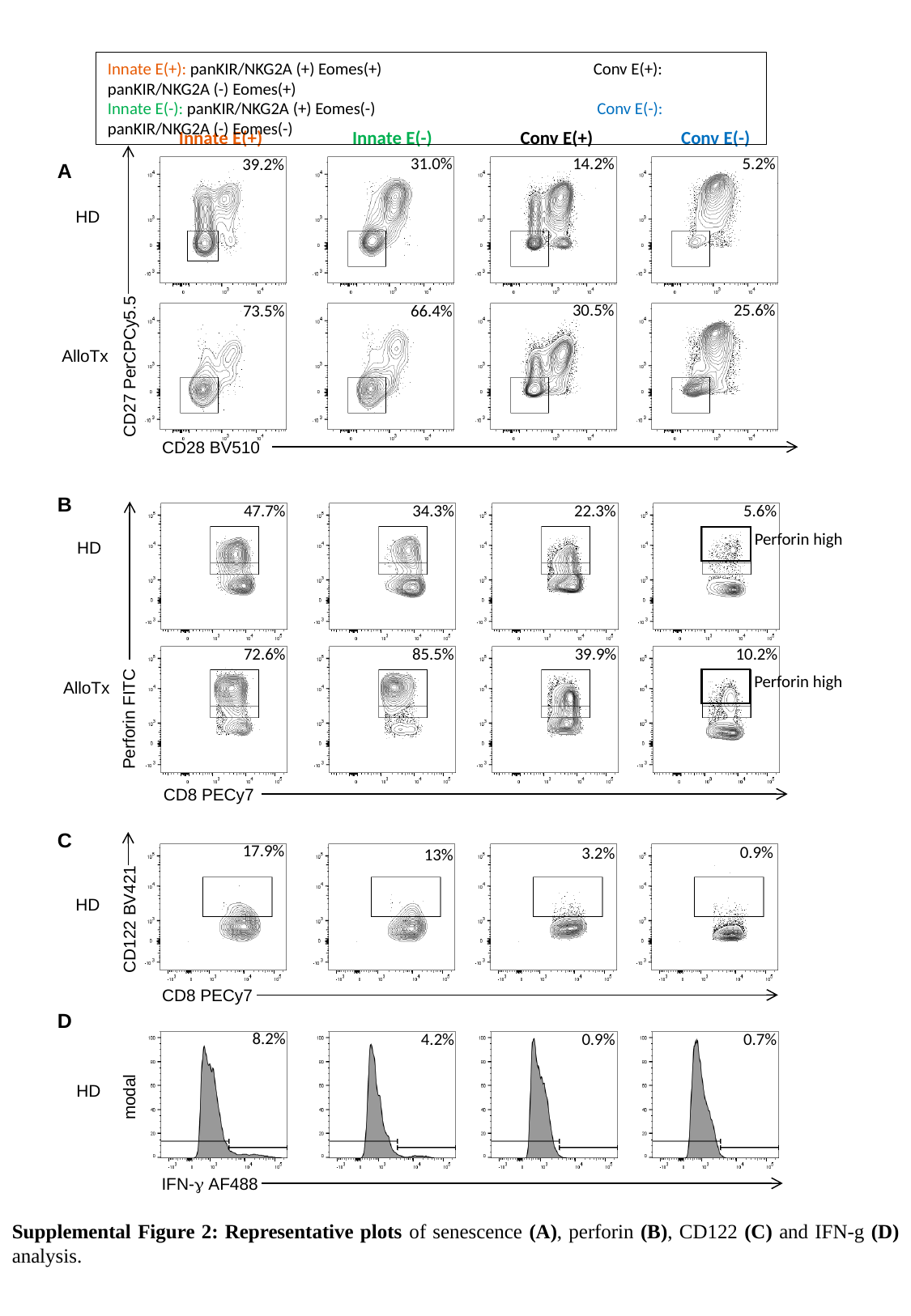

Innate E(+): panKIR/NKG2A (+) Eomes(+)		Conv E(+): panKIR/NKG2A (-) Eomes(+)
Innate E(-): panKIR/NKG2A (+) Eomes(-)		 Conv E(-): panKIR/NKG2A (-) Eomes(-)
Innate E(+)
Innate E(-)
Conv E(+)
Conv E(-)
31.0%
5.2%
14.2%
39.2%
HD
25.6%
30.5%
66.4%
73.5%
AlloTx
CD27 PerCPCy5.5
CD28 BV510
A
B
34.3%
47.7%
5.6%
22.3%
Perforin high
HD
39.9%
72.6%
85.5%
10.2%
Perforin high
AlloTx
Perforin FITC
CD8 PECy7
C
17.9%
0.9%
3.2%
13%
HD
CD122 BV421
CD8 PECy7
D
8.2%
0.9%
0.7%
4.2%
HD
IFN-γ AF488
modal
Supplemental Figure 2: Representative plots of senescence (A), perforin (B), CD122 (C) and IFN-g (D) analysis.

## Slide 3
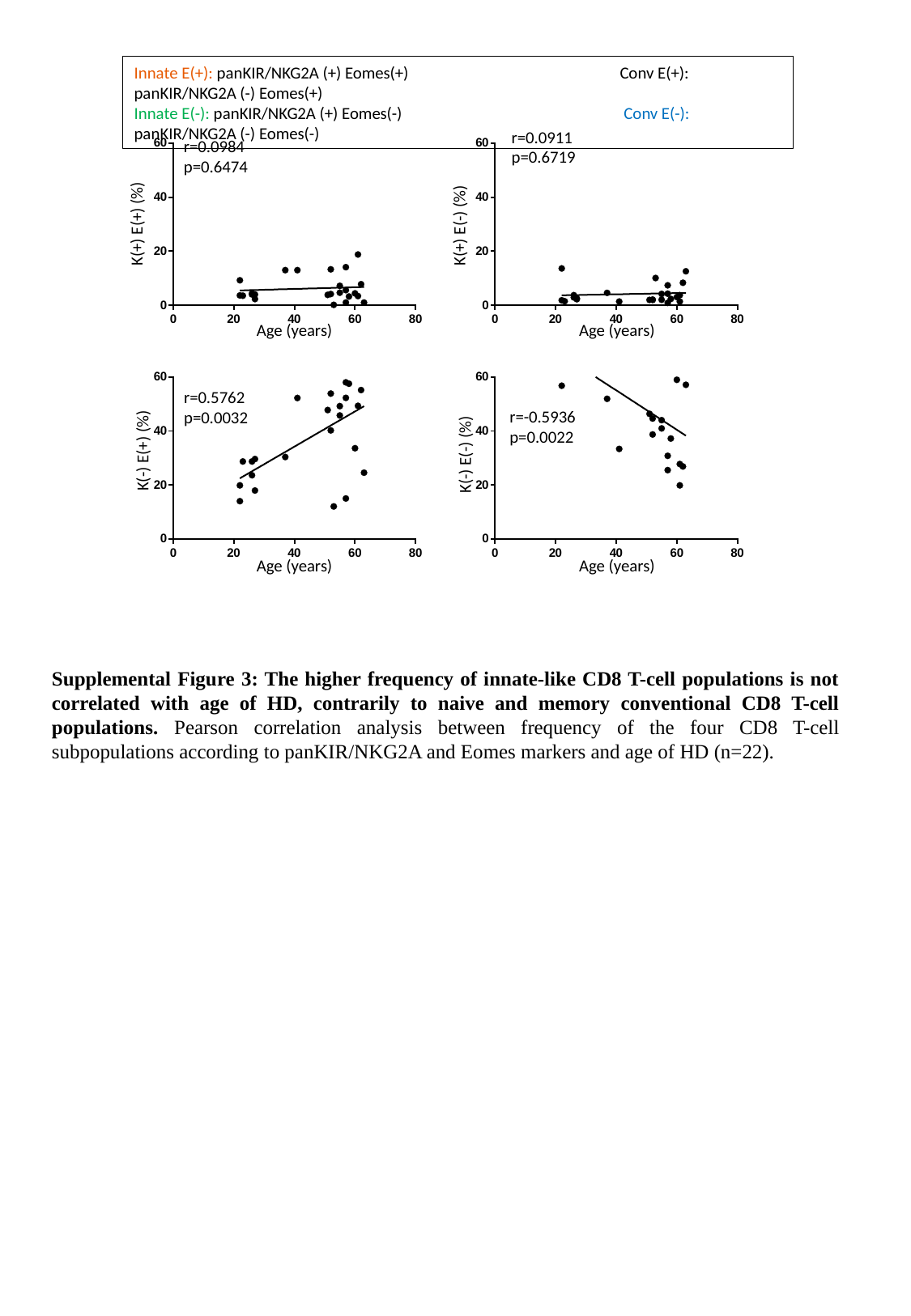

Innate E(+): panKIR/NKG2A (+) Eomes(+)		Conv E(+): panKIR/NKG2A (-) Eomes(+)
Innate E(-): panKIR/NKG2A (+) Eomes(-)		 Conv E(-): panKIR/NKG2A (-) Eomes(-)
r=0.0911
p=0.6719
r=0.0984
p=0.6474
K(+) E(+) (%)
K(+) E(-) (%)
Age (years)
Age (years)
r=0.5762
p=0.0032
r=-0.5936
p=0.0022
K(-) E(+) (%)
K(-) E(-) (%)
Age (years)
Age (years)
Supplemental Figure 3: The higher frequency of innate-like CD8 T-cell populations is not correlated with age of HD, contrarily to naive and memory conventional CD8 T-cell populations. Pearson correlation analysis between frequency of the four CD8 T-cell subpopulations according to panKIR/NKG2A and Eomes markers and age of HD (n=22).

## Slide 4
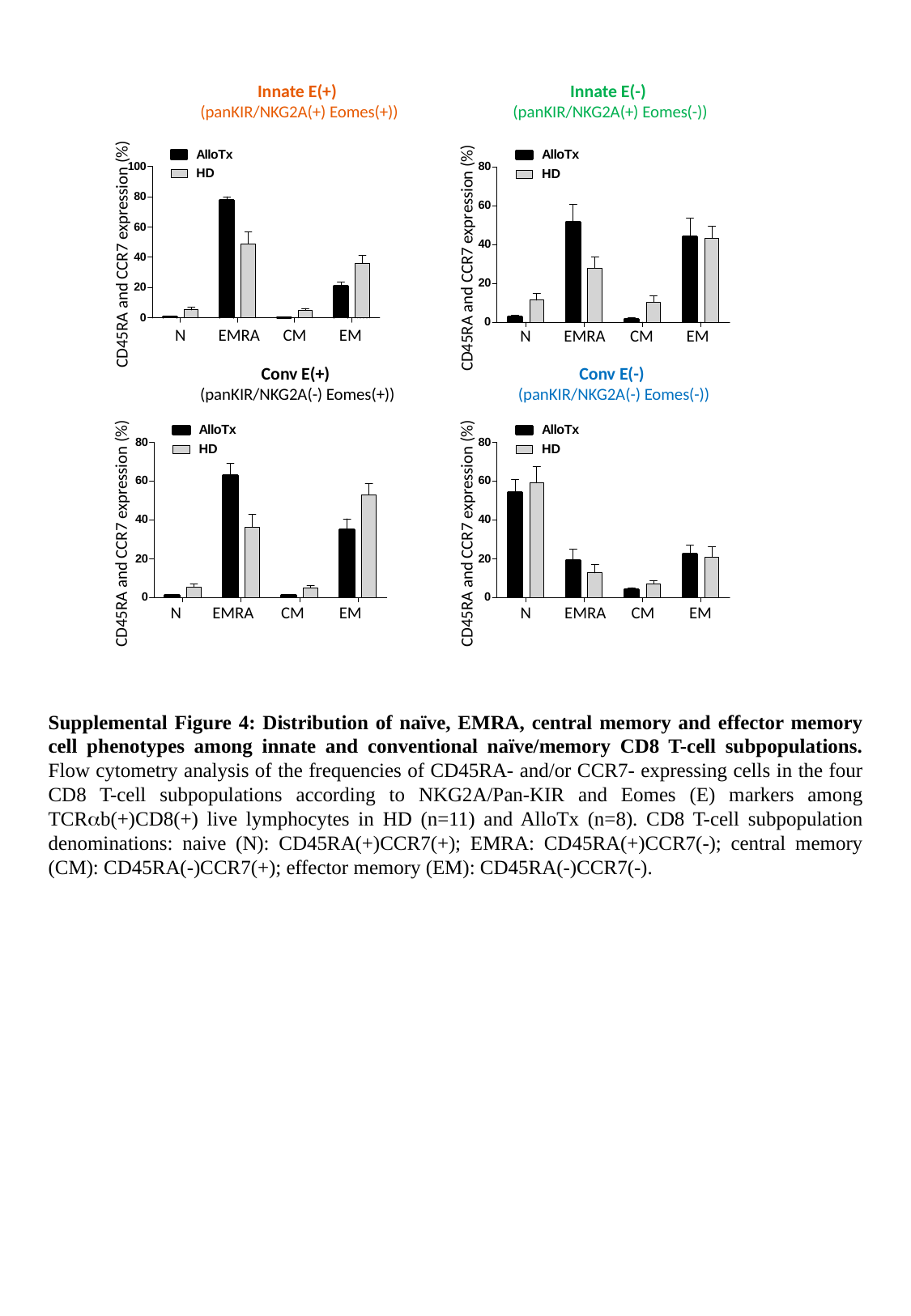

Innate E(+)
(panKIR/NKG2A(+) Eomes(+))
Innate E(-)
(panKIR/NKG2A(+) Eomes(-))
CD45RA and CCR7 expression (%)
CD45RA and CCR7 expression (%)
N
EMRA
CM
EM
N
EMRA
CM
EM
Conv E(+)
(panKIR/NKG2A(-) Eomes(+))
Conv E(-)
(panKIR/NKG2A(-) Eomes(-))
CD45RA and CCR7 expression (%)
CD45RA and CCR7 expression (%)
N
EMRA
CM
EM
N
EMRA
CM
EM
Supplemental Figure 4: Distribution of naïve, EMRA, central memory and effector memory cell phenotypes among innate and conventional naïve/memory CD8 T-cell subpopulations. Flow cytometry analysis of the frequencies of CD45RA- and/or CCR7- expressing cells in the four CD8 T-cell subpopulations according to NKG2A/Pan-KIR and Eomes (E) markers among TCRαb(+)CD8(+) live lymphocytes in HD (n=11) and AlloTx (n=8). CD8 T-cell subpopulation denominations: naive (N): CD45RA(+)CCR7(+); EMRA: CD45RA(+)CCR7(-); central memory (CM): CD45RA(-)CCR7(+); effector memory (EM): CD45RA(-)CCR7(-).

## Slide 5
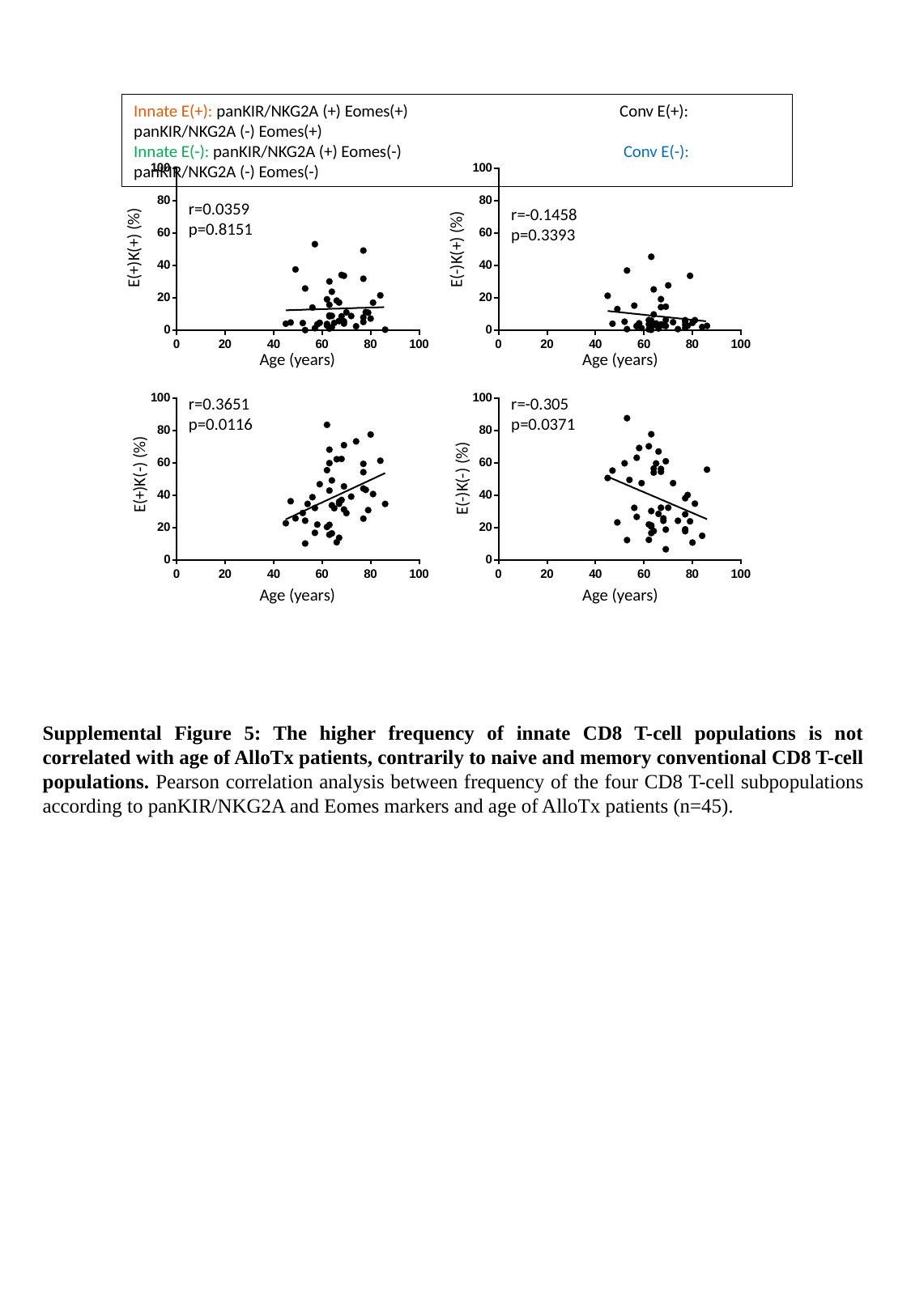

Innate E(+): panKIR/NKG2A (+) Eomes(+)		Conv E(+): panKIR/NKG2A (-) Eomes(+)
Innate E(-): panKIR/NKG2A (+) Eomes(-)		 Conv E(-): panKIR/NKG2A (-) Eomes(-)
r=0.0359
p=0.8151
r=-0.1458
p=0.3393
E(+)K(+) (%)
E(-)K(+) (%)
Age (years)
Age (years)
r=0.3651
p=0.0116
r=-0.305
p=0.0371
E(+)K(-) (%)
E(-)K(-) (%)
Age (years)
Age (years)
Supplemental Figure 5: The higher frequency of innate CD8 T-cell populations is not correlated with age of AlloTx patients, contrarily to naive and memory conventional CD8 T-cell populations. Pearson correlation analysis between frequency of the four CD8 T-cell subpopulations according to panKIR/NKG2A and Eomes markers and age of AlloTx patients (n=45).

## Slide 6
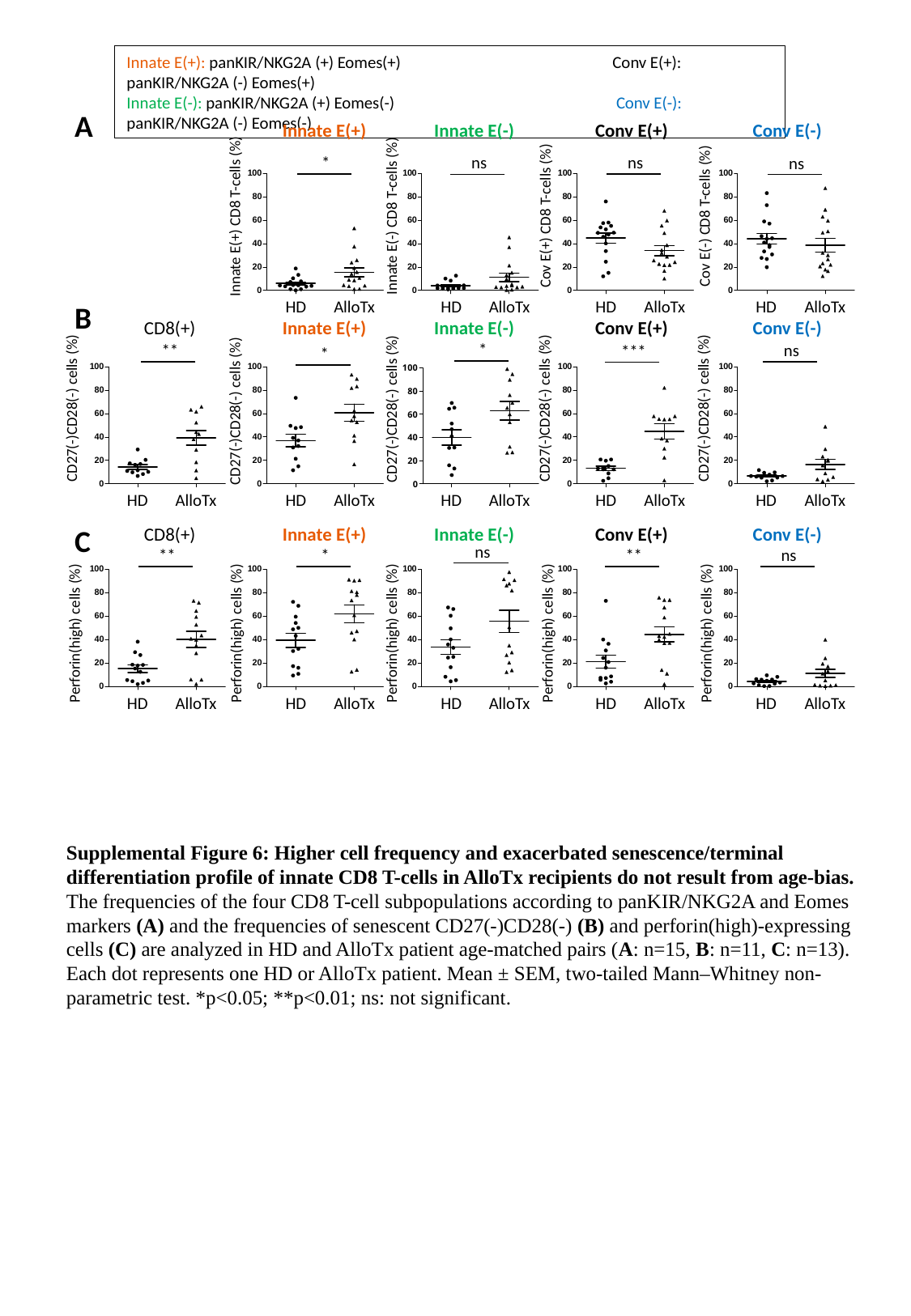

Innate E(+): panKIR/NKG2A (+) Eomes(+)		Conv E(+): panKIR/NKG2A (-) Eomes(+)
Innate E(-): panKIR/NKG2A (+) Eomes(-)		 Conv E(-): panKIR/NKG2A (-) Eomes(-)
A
Innate E(+)
Innate E(-)
Conv E(+)
Conv E(-)
ns
*
ns
ns
Innate E(+) CD8 T-cells (%)
Innate E(-) CD8 T-cells (%)
Cov E(+) CD8 T-cells (%)
Cov E(-) CD8 T-cells (%)
HD
AlloTx
HD
AlloTx
HD
AlloTx
HD
AlloTx
B
CD8(+)
Innate E(+)
Innate E(-)
Conv E(+)
Conv E(-)
*
ns
**
***
*
CD27(-)CD28(-) cells (%)
CD27(-)CD28(-) cells (%)
CD27(-)CD28(-) cells (%)
CD27(-)CD28(-) cells (%)
CD27(-)CD28(-) cells (%)
HD
AlloTx
HD
AlloTx
HD
AlloTx
HD
AlloTx
HD
AlloTx
C
CD8(+)
Innate E(+)
Innate E(-)
Conv E(+)
Conv E(-)
ns
ns
**
*
**
Perforin(high) cells (%)
Perforin(high) cells (%)
Perforin(high) cells (%)
Perforin(high) cells (%)
Perforin(high) cells (%)
HD
AlloTx
HD
AlloTx
HD
AlloTx
HD
AlloTx
HD
AlloTx
Supplemental Figure 6: Higher cell frequency and exacerbated senescence/terminal differentiation profile of innate CD8 T-cells in AlloTx recipients do not result from age-bias. The frequencies of the four CD8 T-cell subpopulations according to panKIR/NKG2A and Eomes markers (A) and the frequencies of senescent CD27(-)CD28(-) (B) and perforin(high)-expressing cells (C) are analyzed in HD and AlloTx patient age-matched pairs (A: n=15, B: n=11, C: n=13). Each dot represents one HD or AlloTx patient. Mean ± SEM, two-tailed Mann–Whitney non-parametric test. *p<0.05; **p<0.01; ns: not significant.

## Slide 7
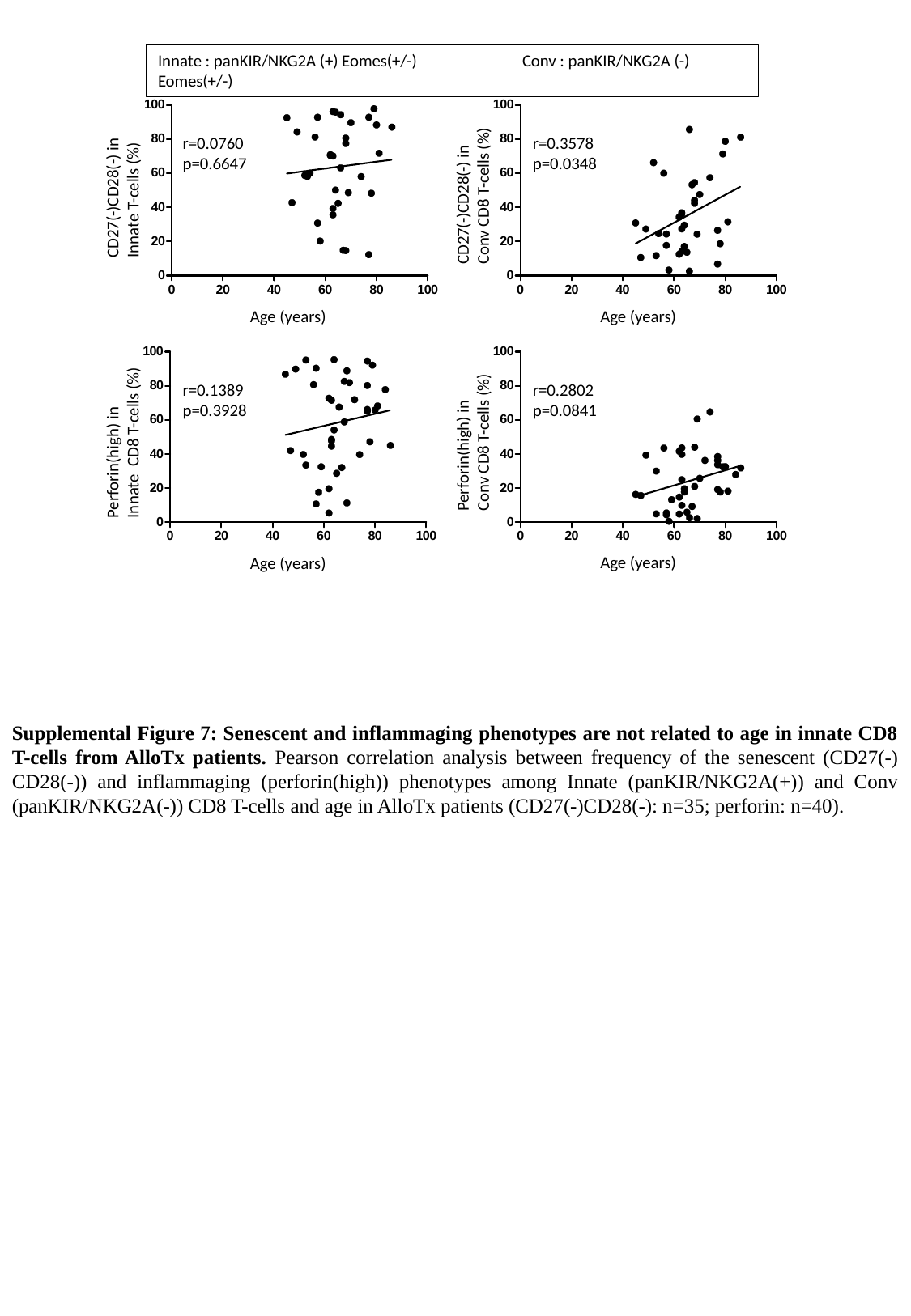

Innate : panKIR/NKG2A (+) Eomes(+/-)	Conv : panKIR/NKG2A (-) Eomes(+/-)
r=0.0760
p=0.6647
CD27(-)CD28(-) in
Innate T-cells (%)
Age (years)
r=0.3578
p=0.0348
CD27(-)CD28(-) in
Conv CD8 T-cells (%)
Age (years)
r=0.1389
p=0.3928
Perforin(high) in
Innate CD8 T-cells (%)
Age (years)
r=0.2802
p=0.0841
Perforin(high) in
Conv CD8 T-cells (%)
Age (years)
Supplemental Figure 7: Senescent and inflammaging phenotypes are not related to age in innate CD8 T-cells from AlloTx patients. Pearson correlation analysis between frequency of the senescent (CD27(-) CD28(-)) and inflammaging (perforin(high)) phenotypes among Innate (panKIR/NKG2A(+)) and Conv (panKIR/NKG2A(-)) CD8 T-cells and age in AlloTx patients (CD27(-)CD28(-): n=35; perforin: n=40).

## Slide 8
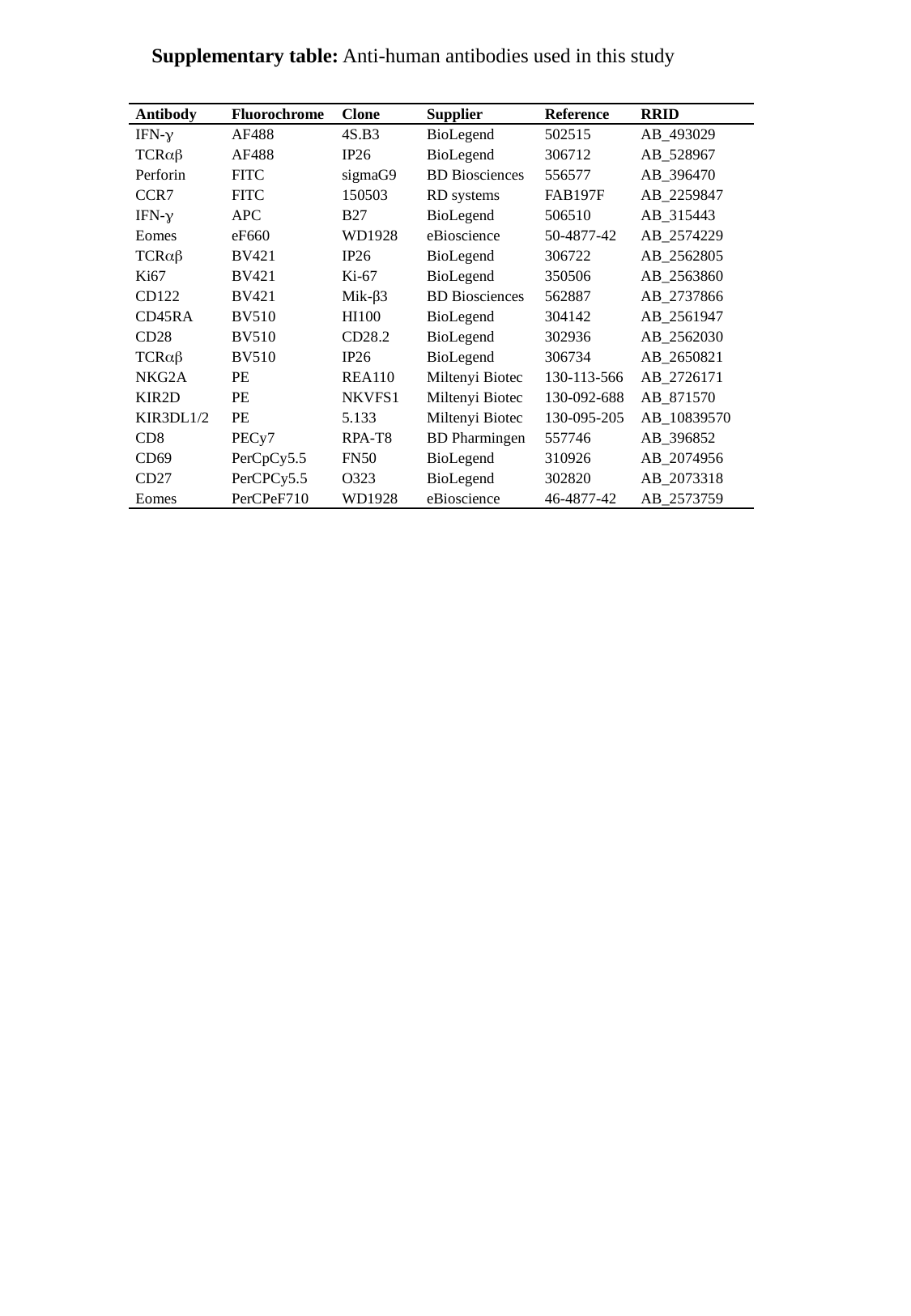

Supplementary table: Anti-human antibodies used in this study
| Antibody | Fluorochrome | Clone | Supplier | Reference | RRID |
| --- | --- | --- | --- | --- | --- |
| IFN-γ | AF488 | 4S.B3 | BioLegend | 502515 | AB\_493029 |
| TCRαβ | AF488 | IP26 | BioLegend | 306712 | AB\_528967 |
| Perforin | FITC | sigmaG9 | BD Biosciences | 556577 | AB\_396470 |
| CCR7 | FITC | 150503 | RD systems | FAB197F | AB\_2259847 |
| IFN-γ | APC | B27 | BioLegend | 506510 | AB\_315443 |
| Eomes | eF660 | WD1928 | eBioscience | 50-4877-42 | AB\_2574229 |
| TCRαβ | BV421 | IP26 | BioLegend | 306722 | AB\_2562805 |
| Ki67 | BV421 | Ki-67 | BioLegend | 350506 | AB\_2563860 |
| CD122 | BV421 | Mik-β3 | BD Biosciences | 562887 | AB\_2737866 |
| CD45RA | BV510 | HI100 | BioLegend | 304142 | AB\_2561947 |
| CD28 | BV510 | CD28.2 | BioLegend | 302936 | AB\_2562030 |
| TCRαβ | BV510 | IP26 | BioLegend | 306734 | AB\_2650821 |
| NKG2A | PE | REA110 | Miltenyi Biotec | 130-113-566 | AB\_2726171 |
| KIR2D | PE | NKVFS1 | Miltenyi Biotec | 130-092-688 | AB\_871570 |
| KIR3DL1/2 | PE | 5.133 | Miltenyi Biotec | 130-095-205 | AB\_10839570 |
| CD8 | PECy7 | RPA-T8 | BD Pharmingen | 557746 | AB\_396852 |
| CD69 | PerCpCy5.5 | FN50 | BioLegend | 310926 | AB\_2074956 |
| CD27 | PerCPCy5.5 | O323 | BioLegend | 302820 | AB\_2073318 |
| Eomes | PerCPeF710 | WD1928 | eBioscience | 46-4877-42 | AB\_2573759 |
